# Supplementary material for: Age-stratified discrete compartment model of the COVID-19 epidemic with application to Switzerland
Source: Sci Rep. 2020 Dec 4;10:21306. doi: 10.1038/s41598-020-77420-4 (PMC7718912; doi:10.1038/s41598-020-77420-4)
Supplement: Supplementary file 1 — Supplementary information. [file 41598_2020_77420_MOESM1_ESM.pdf]

Supplementary Information for

Age-stratified discrete compartment model of the COVID-19 epidemic with application to Switzerland

by

*Fadoua Balabdaoui and Dirk Mohr**

F. Balabdaoui[1], D. Mohr[2]

[1]Seminar of Statistics, Department of Mathematics, Swiss Federal Institute of Technology (ETH), Rämistrasse 101, Zurich, 8092, Switzerland

[2]Department of Mechanical and Process Engineering, Swiss Federal Institute of Technology (ETH), Tannenstrasse 3, Zurich, 8092, Switzerland

*corresponding author: Dirk Mohr, dmohr@ethz.ch

Supplementary notes

Probability of death in sub-compartments

For the compartments C (self-isolation), H (middle-care unit) and Q (intensive care unit), we define a fatality ratio as a function of age and the duration in the compartment. The time dependency is modeled through a bump function. For a compartment with a transit time of T , the time dependent fatality ratio reads

$$p(t,a) = \frac{p_0(a)}{0.4439} \exp\left(\frac{1}{[t/(T/2)+1]^2 - 1} \right) \quad \text{for} \quad -T/2 \le t \le T/2$$

with the age dependent parameter $p_0 = p_0(a)$. The continuous function is converted into a discrete version providing the fatality ratio per sub-compartment k using the expression

$$p^{(k)}(a) = \frac{1}{\Delta t} \int_{(k-1)\Delta t}^{k\Delta t} p(a,t)dt \quad \text{with} \quad k = 1,..,T/\Delta t$$

with $\Delta t = 1day$. The overall probabilities of death in the above compartments are provided in Supplementary Table 1. The transit times corresponds to the number of sub-compartments (Fig. 1), i.e. 6 days for self-isolation, 7 days for MCU and 8 days for ICU. A bar plot of the daily risks of death in ICU for age-groups #9 to #17 is given in Supplementary Fig. 7.

Sample size needed for random testing

Despite the availability of a wealth of data on the hospitalizations and deaths, data on the number of past infected in a random population is still missing. With the emergence of reliable serological tests for detecting past infections, the testing of a randomly selected sample of the Swiss population may be envisioned to further validate the estimates from epidemiological modeling.

In the sequel, we estimate the size of the random sample required to validate the current fraction η of the infected individual in the total population with reasonable accuracy. Standard Gaussian approximation of the estimated proportion $\tilde{\eta}_N$ obtained from the outcome of N independent tests implies that with asymptotic probability of 95% the proportion of infected belongs to the confidence interval $[\tilde{\eta}_N - L/2, \tilde{\eta}_N + L/2]$ with

$$L = \frac{2z_{0.975}}{\sqrt{N}} \sqrt{\eta(1-\eta)}$$

where $z_{0.975} = 1.96$ denotes the 97.5% upper quantile of a standard Gaussian distribution. Measuring a fraction of infection of about $\eta = 0.1$ (which corresponds to our model estimate for early June) with an accuracy of ± 0.005 $(\pm 5\%)$ would hence require minimum sample size of $N_{\min} = 13,830$. An accuracy of $\pm 10\%$ would already be achieved with $N_{\min} = 3,457$.

Multi-variate effect of the probabilities of transmission

To complement the results of the relaxation scenarios discussed in the main document, we also present the results from a parametric study on the effect of the rate of transmission at different locations. The baseline scenario for our computations is that presented in Fig. 7. In particular, the history until June 8 is the same. After June 8, we still consider that 30% of the workforce stays in home office $(\phi_{work} = 0.65)$, a reduced rate of contacts at other locations $(\phi_{other} = 0.7)$ and open schools $(\phi_{school} = 1)$. However, the probability of transmission at these three locations is varied from 0.25 to 1.0 with steps of 0.25. The resulting matrix of all 64 combinations of β^*_{other}, β^*_{work} and β^*_{school} is given in Supplementary Table 2 along with the main model outputs (second peak in ICU, fraction of total population infected, and fatalities by Dec 2020). For 44 scenarios, the number of fatalities exceeds that of the baseline scenario. Unless a probability of transmission of 0.25 is reached for at least one location, the number of fatalities will exceed 7'000. The analysis of the linear correlation coefficients $\rho_.$ between the probabilities of transmission at different locations and the number of fatalities ($\rho_{other} = 0.70$, $\rho_{work} = 0.50$ and $\rho_{school} = 0.48$) revealed that the protection at all three locations is crucial to maintain a low number of fatalities after relaxing the lockdown.

Effect of transmission through self-isolated individuals

All results presented in the main text are obtained assuming 'perfect self-isolation', i.e. the individuals in the compartment C (symptomatic in self-isolation, Fig. 1) are not contributing to the infection dynamics. To gain some insight into the impact of that strong assumption, we repeated the simulations for the worst scenario (no special caution at school, Fig. 7) assuming that a small fraction γ of the individuals in self-isolation is as infectious as the individuals in compartment B (symptomatic and infectious, Fig. 1). The results for $\gamma = 5\%$ and 10% are depicted as orange and red curves next to the baseline solution ($\gamma = 0$, black curve) in Supplementary Fig. 9. Note that the basis probability of transmission β_0 has been readjusted

for each model such as to match the peak of the first wave. The results reveal that the reduced effectiveness of self-isolation would result in an earlier arrival and an increased intensity of the second wave. For $\gamma = 10\%$, we observe a 17% increase in the peak hospital need and an increase of 9% in the fatalities by the end of 2020.

Advanced model accounting for presymptomatic transmission

There is growing evidence in the recent literature that the viral shedding is already very high a few days before the onset of symptoms. Based on the analysis of He et al. (2020) an advanced model is built where we assume that individuals in the sub-compartments E4 and E5 (last two days before the onset of symptoms, Fig. 1) are as infectious as the asymptotic infectious individuals in sub-compartments A1 and A2. Furthermore, it is assumed that the infectiousness of the individuals in compartment A decreases linearly over time, from 100% in sub-compartment A2 to 14% in A8 (whose extrapolatation would lead to 0% for individuals in a hypothetical A9). The transmission probabilities of the advanced model are then calibrated based on the data for hospitalization from March 1 to June 20. Supplementary Fig. 10 compares the predictions of the advanced model (with presymptomatic transmission) and the baseline model for the worst case scenario considered in Fig. 7 (no special caution when reopening schools). The comparison of the black and orange curves reveals only little differences for all categories. This observation suggests that the exact transmission dynamics play only a subordinate role as far as the prediction of the second wave is concerned provided that the model is calibrated based on the results from the first wave. On a side note, it is recalled that the baseline model (black curve) had only been calibrated on data until April 20. The good agreement of the model predictions with the data from April 21 to June 20 may thus be seen as a partial validation of the proposed model.

References

He, X. *et al.* Temporal dynamics in viral shedding and transmissibility of COVID 19, *Nat. Med.* **26**, 672-675; 10.1038/s41591-020-0869-5 (2020).

Supplementary Tables

Supplementary Table 1 Model paramaters (discrete probability functions) for COVID-19 in Switzerland

Probabilities	age-groups 1 to 17																
	0-4	5-9	10-14	15-19	20-24	25-29	30-34	35-39	40-44	45-49	50-54	55-59	60-64	65-69	70-74	75-79	>80
Hospitalization of symtomatic	0.2%	0.2%	0.2%	0.2%	0.7%	0.7%	0.7%	0.7%	0.8%	1.2%	2.5%	3.9%	8.0%	12.3%	18.0%	27.1%	40.0%
Transfer from MCU to ICU	-	-	-	1.0%	4.2%	8.5%	12.7%	17.0%	21.2%	25.5%	25.5%	29.7%	29.7%	29.7%	29.7%	15.6%	7.8%
Death in self-isolation	-	-	-	-	-	-	-	-	-	-	-	-	-	-	-	2.3%	7.3%
Death in MCU	-	-	-	-	-	-	-	-	-	-	-	-	-	-	-	-	18.0%
Death in ICU	0.9%	0.9%	0.9%	0.9%	0.9%	0.9%	0.9%	0.9%	0.9%	0.9%	5.3%	10.9%	20.5%	30.1%	41.8%	57.7%	64.2%

Supplementary Table 2 Multivariate effect of the probability of transmission

scenario	Probability of transmission at other locations	work	school	Fraction of population infected by Dec 2020	Second peak in ICU [per 100,000]	Fatalities by Dec 2020
1	1	1	1	60.0	2,048	16,196
2	1	1	0.75	57.5	1,732	15,543
3	1	0.75	1	55.3	1,689	15,103
4	1	1	0.5	54.5	1,425	14,737
5	1	0.75	0.75	52.3	1,356	14,279
6	0.75	1	1	55.4	1,585	13,925
7	1	0.5	1	50.0	1,365	13,811
8	1	1	0.25	50.9	1,149	13,744
9	1	0.75	0.5	48.4	1,034	13,206
10	0.75	1	0.75	52.4	1,281	13,175
11	0.75	0.75	1	50.1	1,267	12,767
12	1	0.5	0.75	46.1	1,022	12,750
13	1	0.25	1	44.2	1,089	12,380
14	0.75	1	0.5	48.7	992	12,219
15	0.75	0.75	0.75	46.3	953	11,801
16	1	0.75	0.25	43.4	751	11,772
17	0.5	1	1	50.3	1,181	11,741
18	0.75	0.5	1	44.2	995	11,453
19	1	0.5	0.5	40.9	695	11,251
20	1	0.25	0.75	39.4	748	11,036
21	0.75	1	0.25	44.1	742	10,999
22	0.5	1	0.75	46.7	896	10,881
23	0.5	0.75	1	44.5	914	10,568
24	0.75	0.75	0.5	41.3	659	10,468
25	0.75	0.5	0.75	39.5	681	10,211
26	0.75	0.25	1	38.4	777	10,105
27	0.5	1	0.5	41.9	634	9,728
28	0.25	1	1	44.9	844	9,707
29	0.5	0.75	0.75	39.8	627	9,447
30	0.5	0.5	1	38.4	699	9,323
31	1	0.5	0.25	33.7	423	9,097
32	1	0.25	0.5	32.7	435	9,020
33	0.25	1	0.75	40.4	586	8,723
34	0.75	0.75	0.25	34.6	417	8,630
35	0.75	0.25	0.75	32.8	476	8,597
36	0.25	0.75	1	38.7	632	8,589
37	0.75	0.5	0.5	32.8	397	8,366
38	0.5	1	0.25	35.9	422	8,218
39	0.5	0.25	1	32.9	536	8,164
40	0.5	0.5	0.75	32.8	424	7,925
41	0.5	0.75	0.5	33.3	374	7,816
42	0.25	0.5	1	32.9	472	7,510
43	0.25	1	0.5	34.3	362	7,336
44	0.25	0.75	0.75	33.1	383	7,327
Reference (Fig. 7)	0.3	0.3	1	29.9	411	7,062
45	0.25	0.25	1	28.1	359	6,607
46	0.5	0.25	0.75	26.6	286	6,584
47	0.75	0.25	0.5	24.7	229	6,379
48	1	0.25	0.25	23.6	208	6,344
49	0.25	0.5	0.75	26.4	247	6,040
50	0.75	0.5	0.25	24.0	196	6,007
51	0.5	0.5	0.5	24.6	203	5,901
52	0.5	0.75	0.25	25.0	193	5,797
53	0.25	1	0.25	26.6	201	5,666
54	0.25	0.75	0.5	25.0	187	5,536
55	0.25	0.25	0.75	21.5	166	5,070
56	0.5	0.25	0.5	18.5	122	4,538
57	0.75	0.25	0.25	16.6	115	4,239
58	0.25	0.5	0.5	18.2	107	4,212
59	0.5	0.5	0.25	16.8	104	4,046
60	0.25	0.75	0.25	17.4	94	3,935
61	0.25	0.25	0.5	14.6	84	3,497
62	0.5	0.25	0.25	13.2	89	3,258
63	0.25	0.5	0.25	13.2	81	3,142
64	0.25	0.25	0.25	11.7	75	2,842

Supplementary Figures

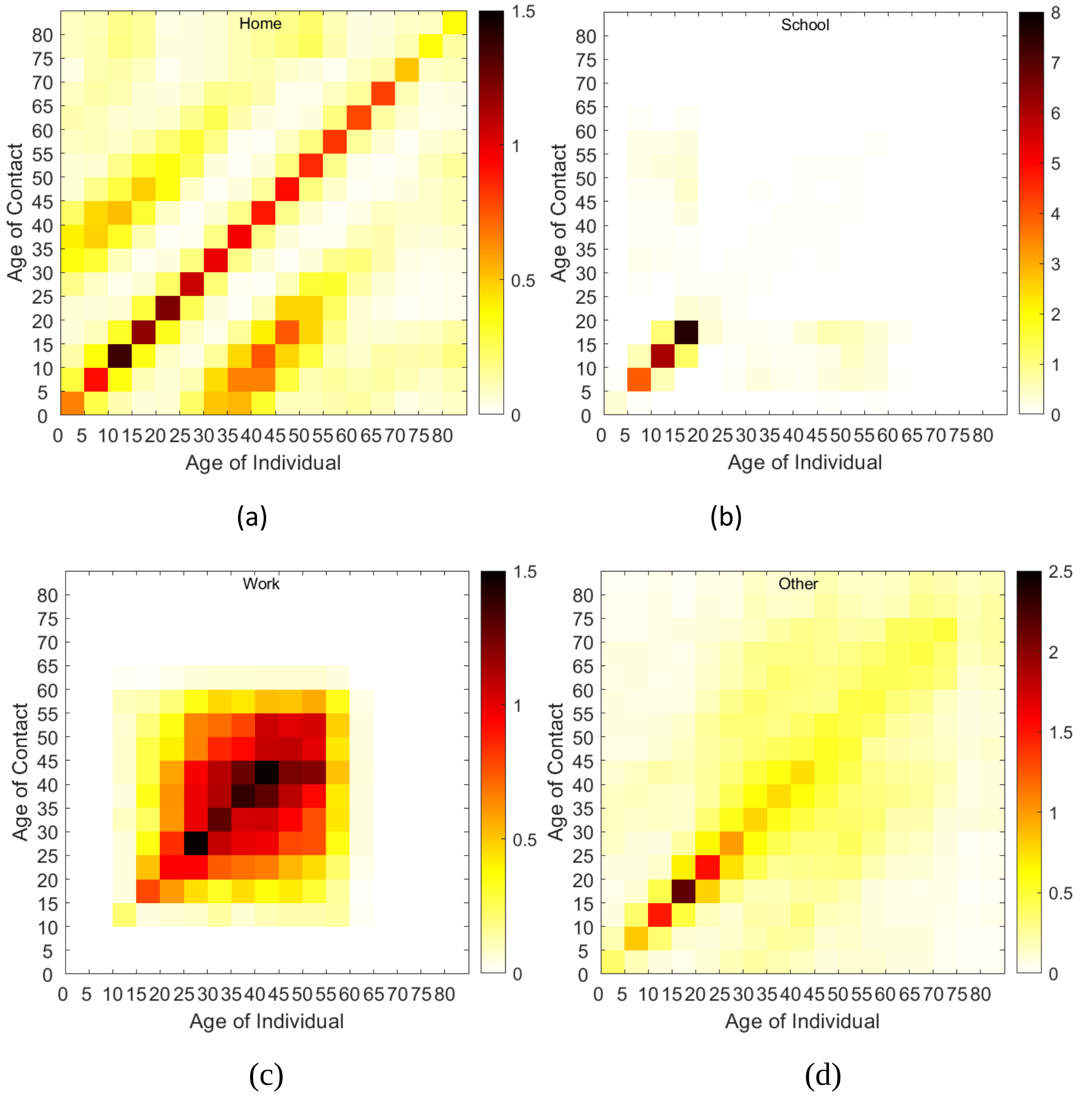

Supplementary Fig. 1. Contact maps for Switzerland as adopted from Prem et al. (2017) for contacts (a) at home, (b) at school, (c) at work, (d) at other locations.

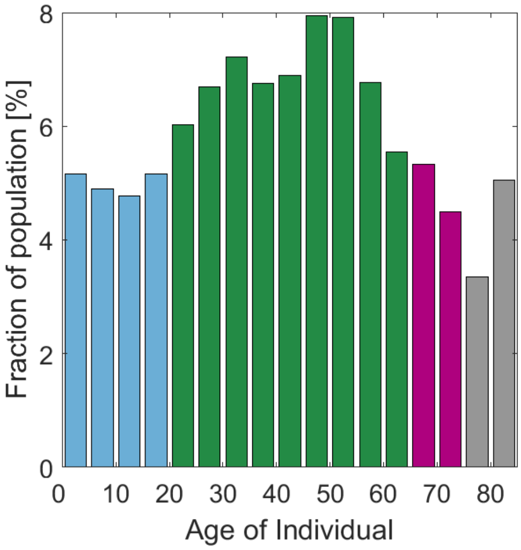

Supplementary Fig. 2. Age distribution in Switzerland in 2016 after www.populationpyramid.net (total population: 8,379,914).

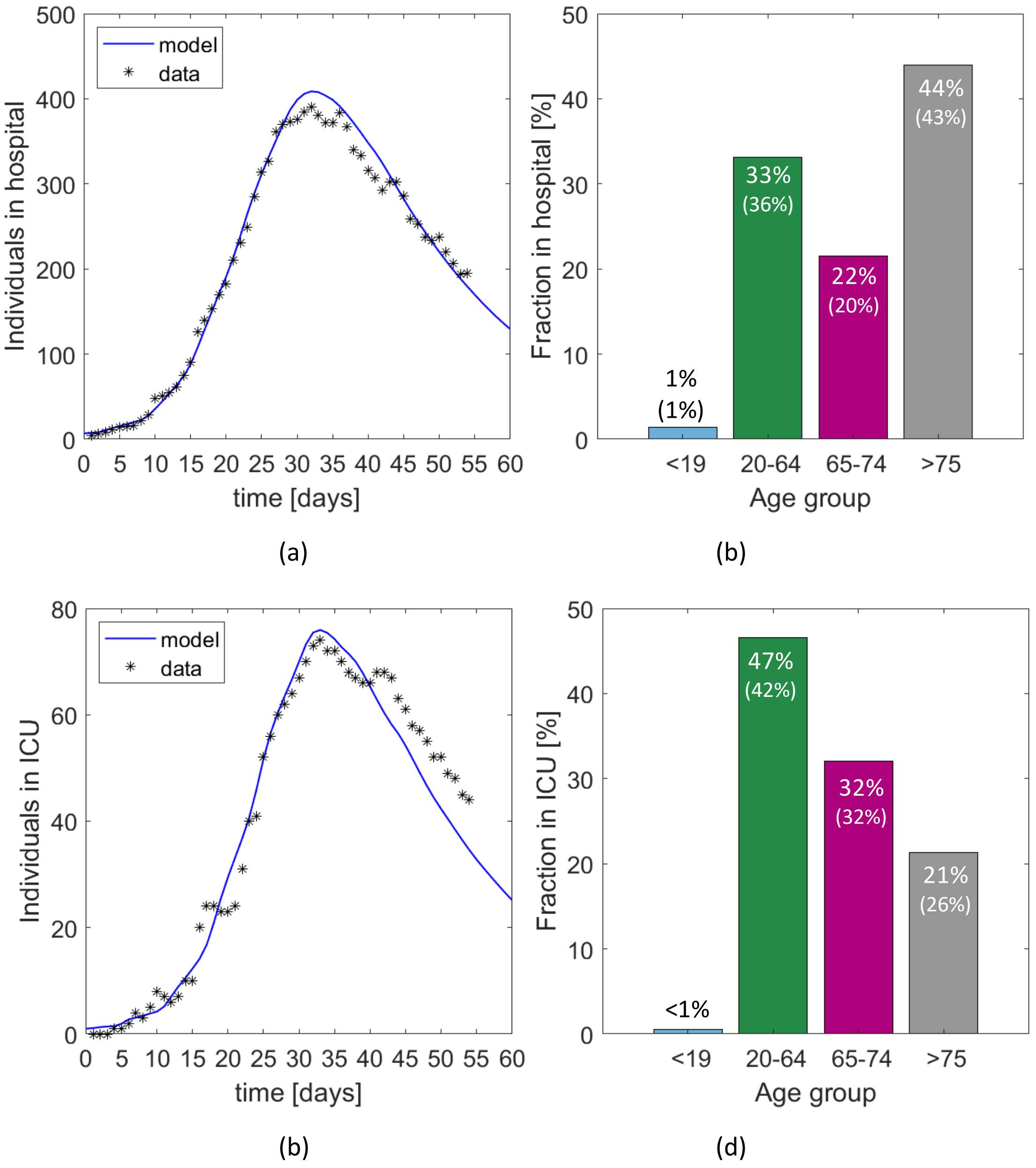

Supplementary Fig. 3. Model identification and validation for the canton Vaud. (a) history of individuals in hospital (MCU and ICU), (b) predicted age-distribution in hospital, (c) history of individuals in ICU, (d) predicted age-distribution in ICU. The values in parentheses in (b) and (d) are data published by the canton Vaud for April 2020.

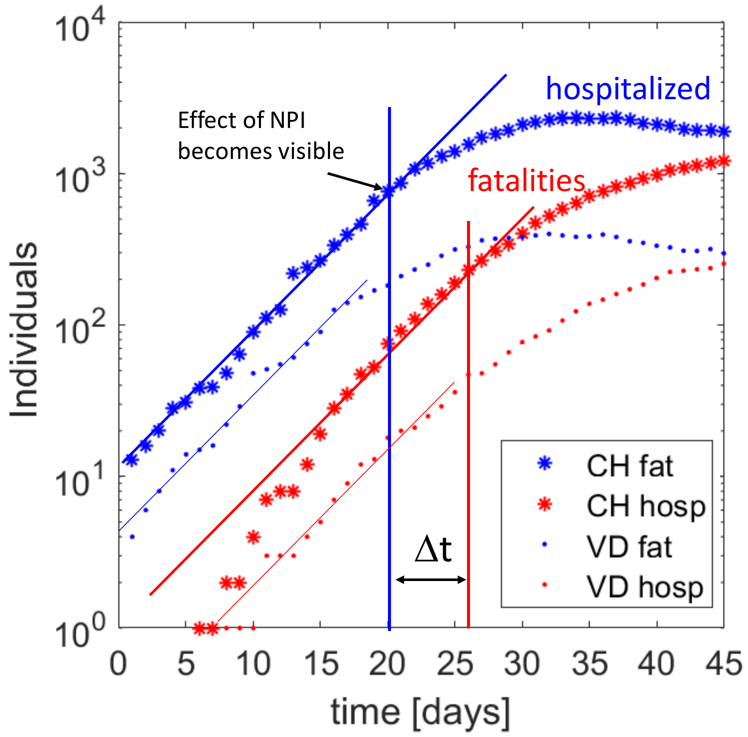

Supplementary Fig. 4. Logarithmic plot of the histories of currently hospitalized and accumulated deceased in Switzerland and the canton Vaud. Day 1 on the time axis corresponds to March 1, 2020.

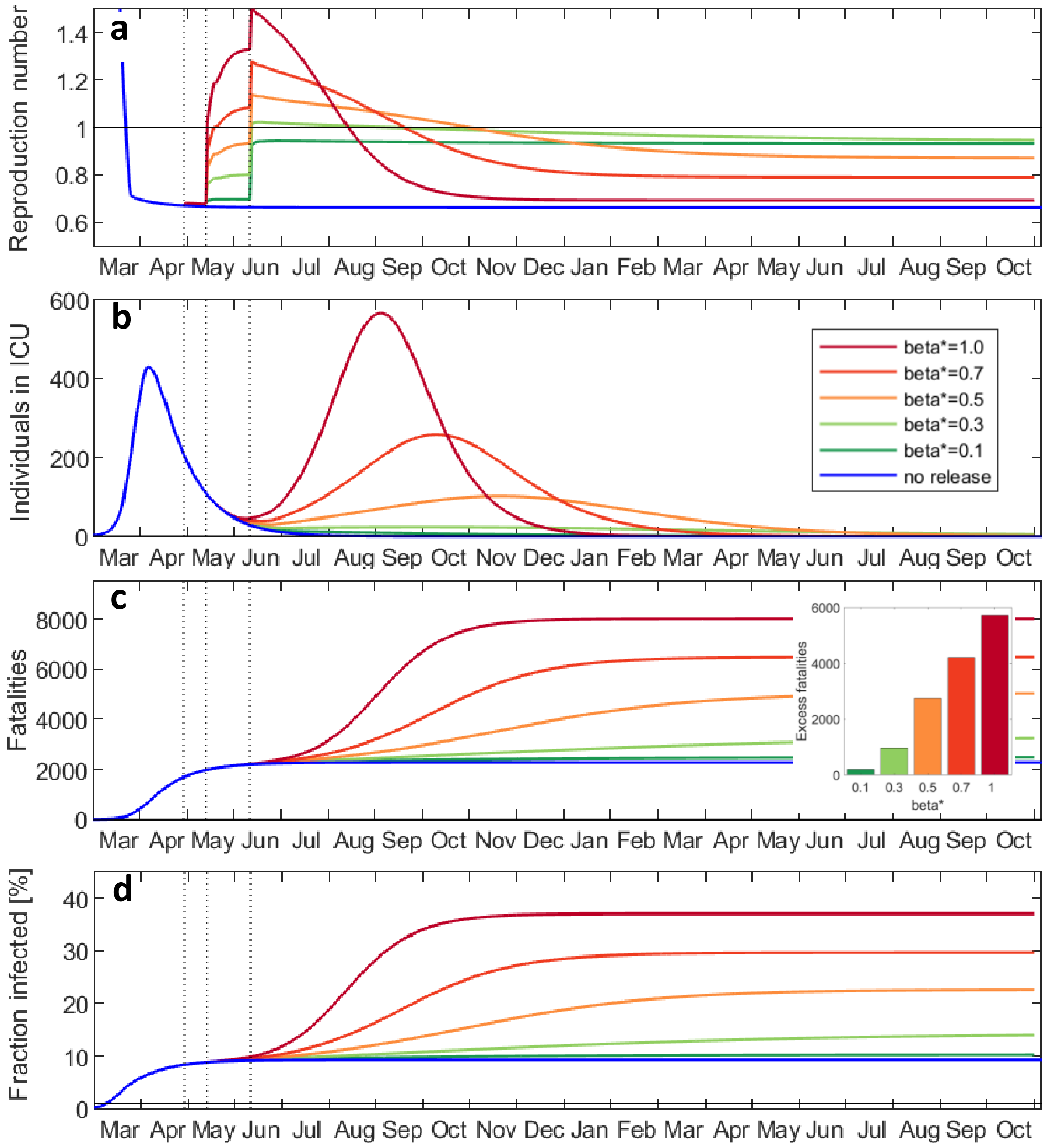

Supplementary Fig 5. Effect of special caution at school (for all age groups) on the evolution of the epidemic under the assumption of no home office (95% of workforce back). Histories of (a) average reproduction number, (b) individuals in ICU, and (c) accumulated fatalities. The factor β^* represents the reduction of the probability of transmission at school.

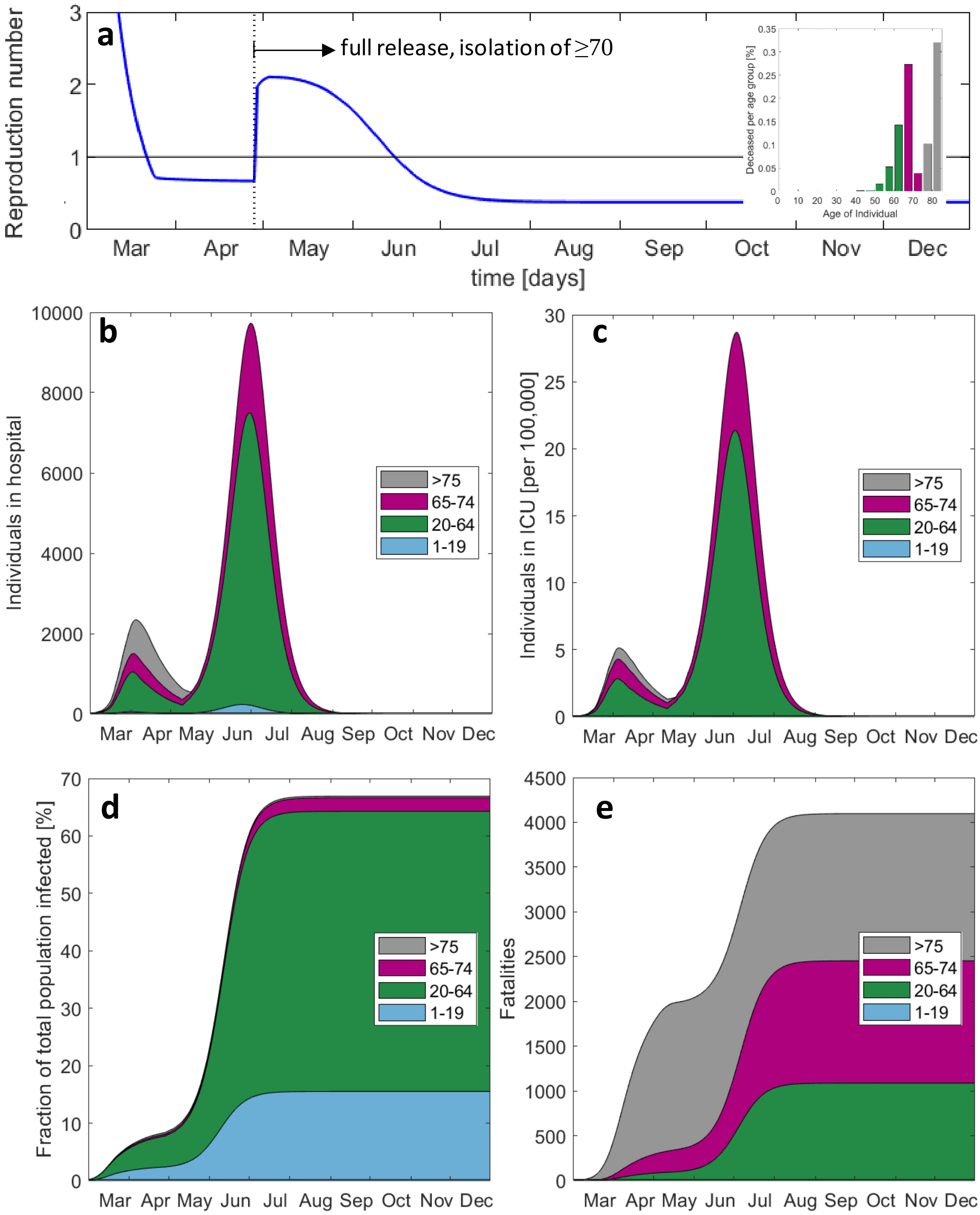

Supplementary Fig. 6. Predicted evolution of the epidemic after full release of all measures after isolating individuals aged 70 years upwards. (a) average reproduction number, (b) number of individuals in hospital, (c) number of individuals in ICU, (d) total cases, (e) accumulated fatalities.

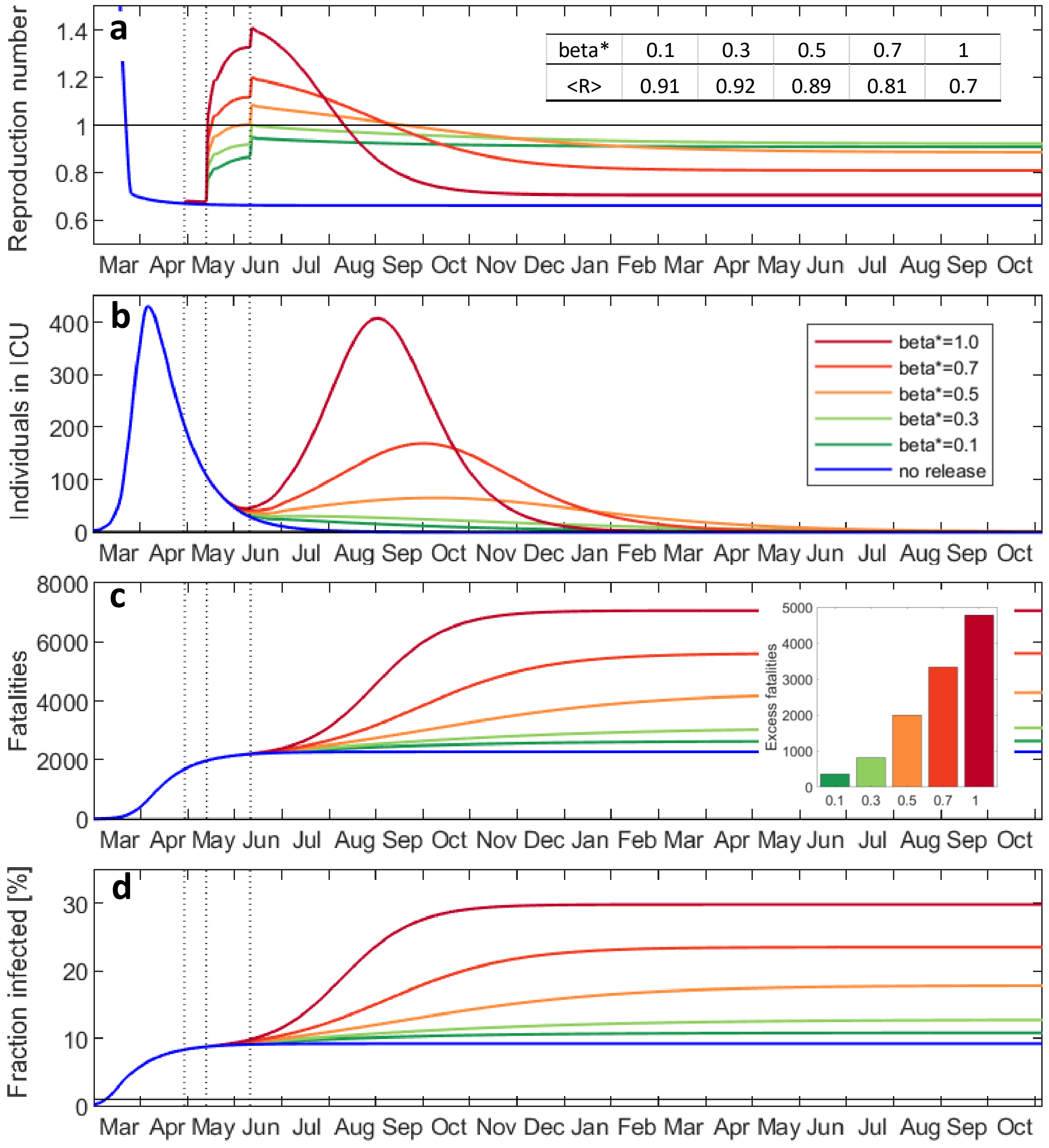

Supplementary Fig 7. Effect of protective measures at school (for those aged 10 upwards while excluding those younger than 10 years) on the evolution of the epidemic under the assumption of 30% home office. Histories of (a) average reproduction number, (b) individuals in ICU, (c) accumulated fatalities, and (d) fraction of the total population infected. The factor β* represents the reduction of the probability of transmission at school for those aged 10 upwards. The table insert in (a) denotes the average reproduction number in October 2021.The insert in (c) shows the death toll (fatalities in excess of result for no release) for relaxing the soft lock-down as a function of β*.

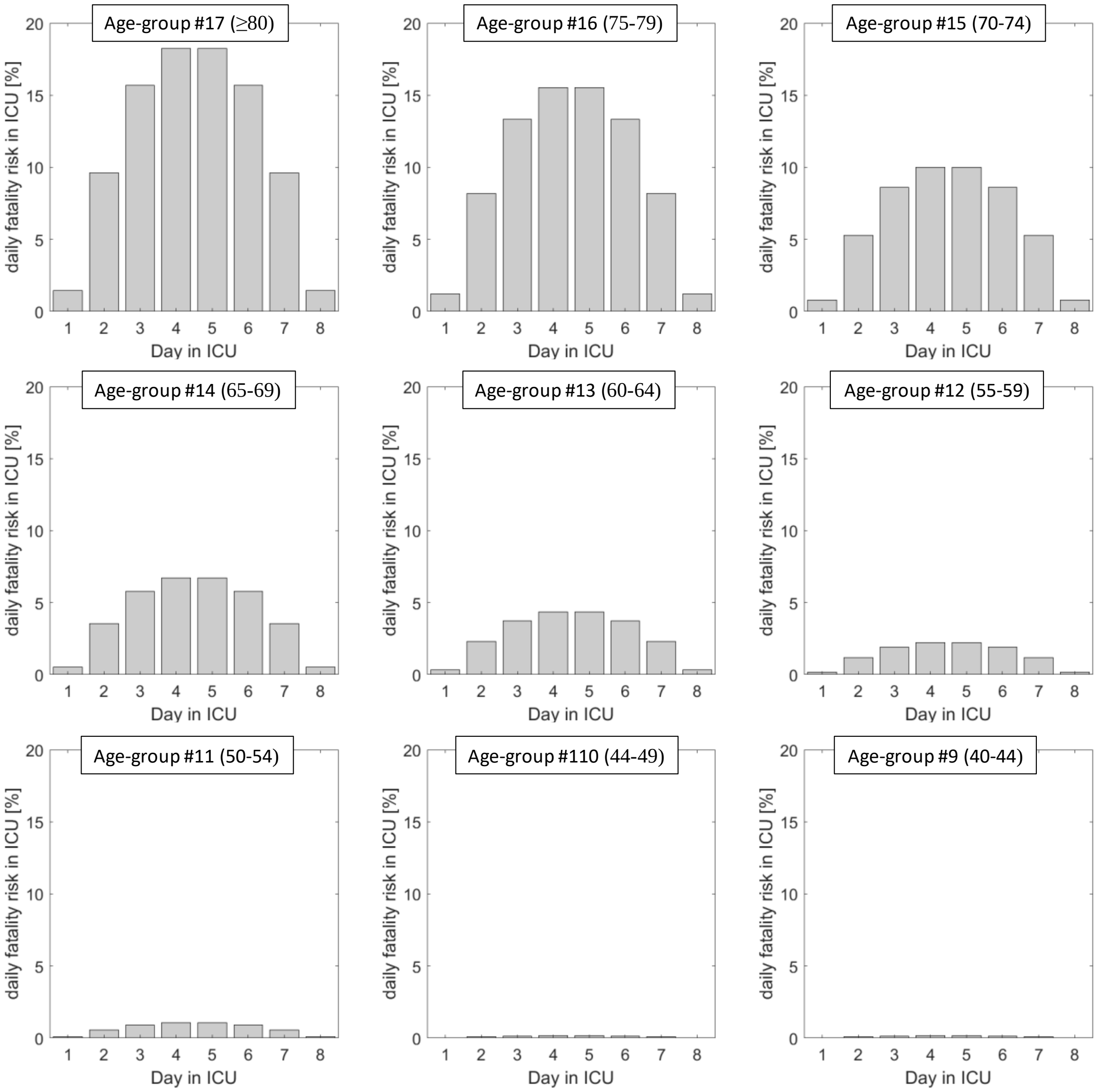

Supplementary Fig 8. Age-group dependent daily probability of death in ICU.

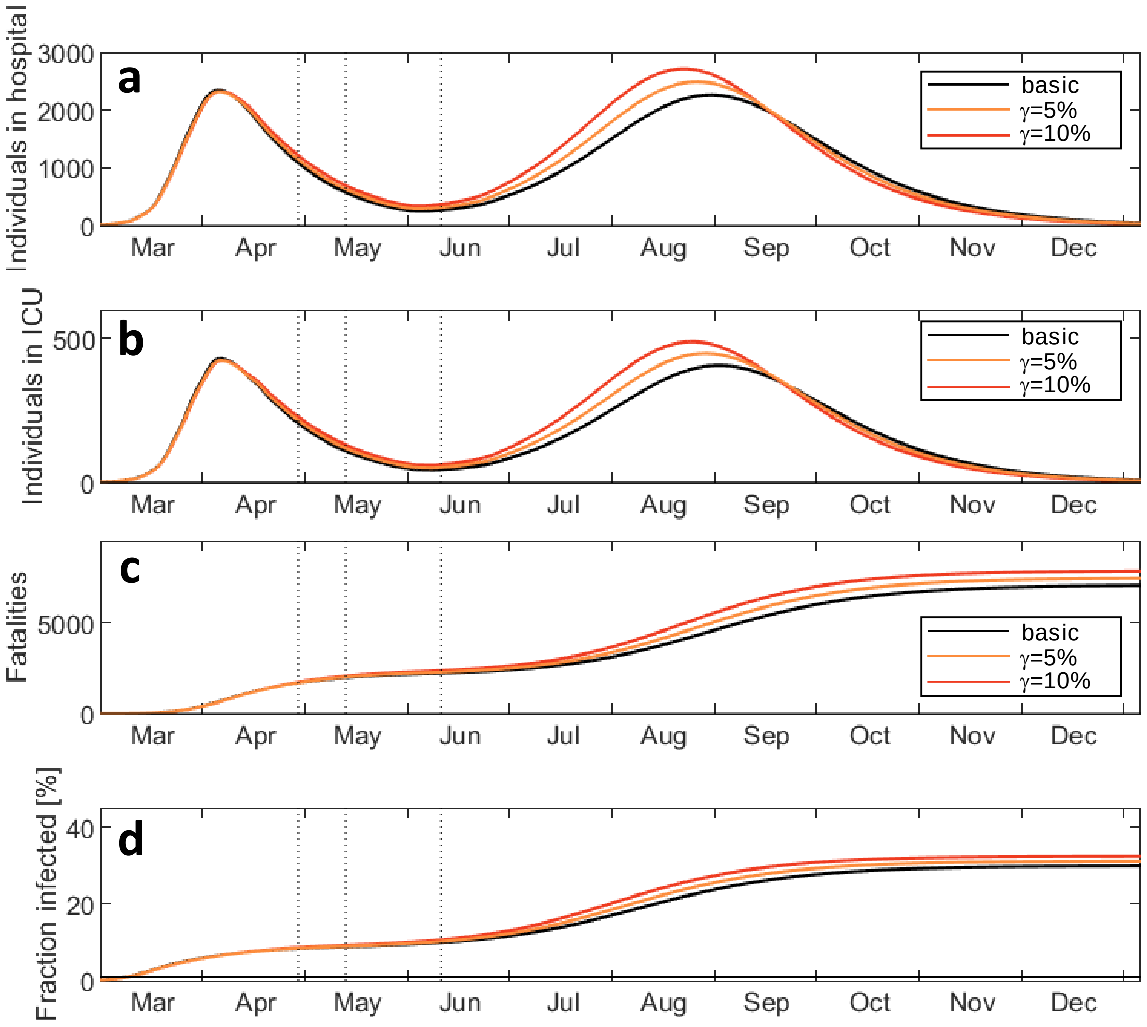

Supplementary Fig 9. Effect of incomplete self-isolation. Prediction of the evolution of the epidemic after step-wise release of measures including school reopening without special caution using the basic model (same as in Fig. 7) and an extended model assuming 5% and 10% of the self-isolated individuals remaining infectious and in contact with susceptible individuals. Histories of (a) individuals in hospital (MCU and ICU), (b) individuals in ICU, (c) accumulated fatalities, and (d) fraction of infected individuals in the total population.

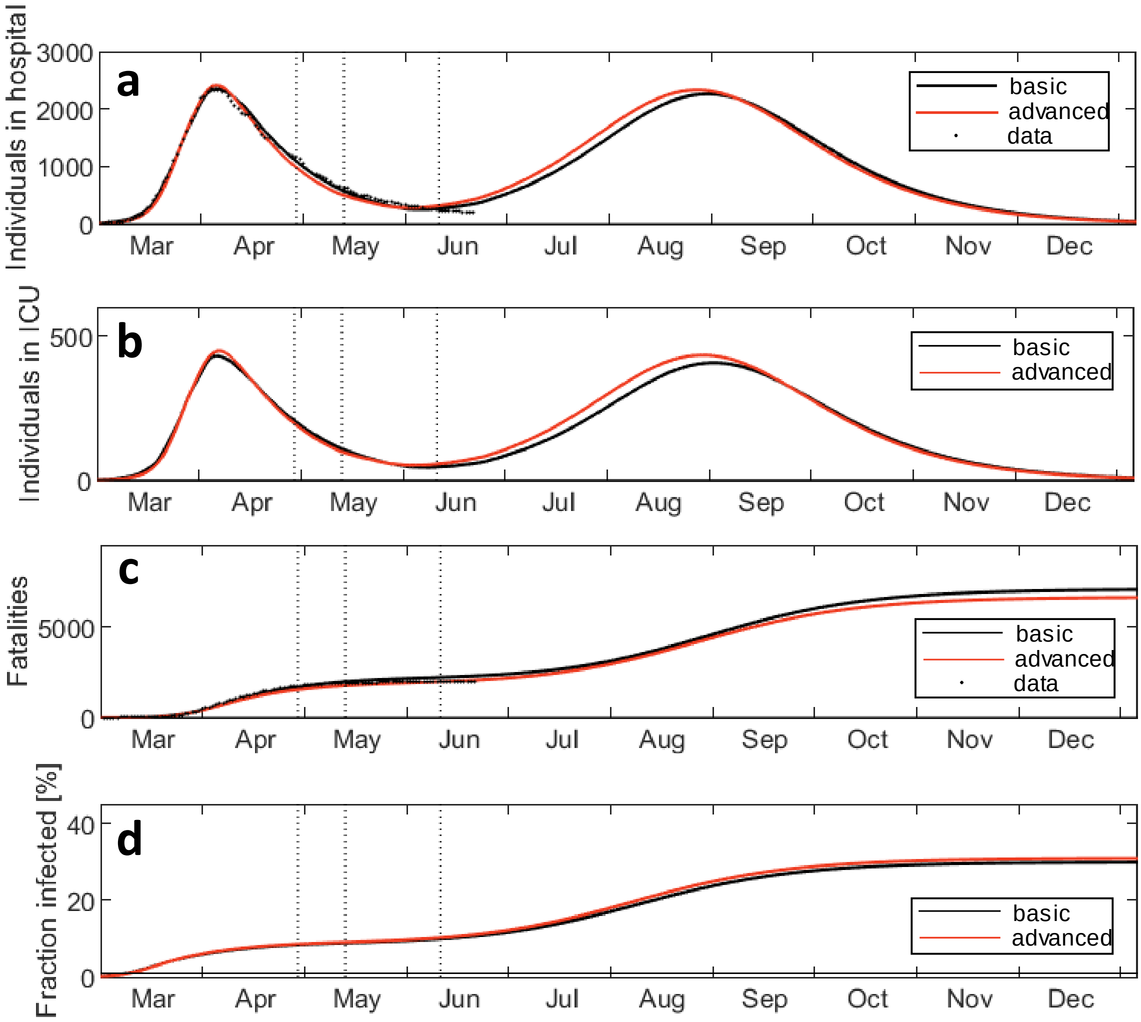

Supplementary Fig 10. Effect of pre-symptomatic infectiousness. Prediction of the evolution of the epidemic after step-wise release of measures including school reopening without special caution using the basic model (same as in Fig. 7) and an advanced model assuming infectiousness already 2 days before the onset of symptoms. Both models are calibrated based on the first wave of hospitalizations (data from Mar 1 to Apr 20 used for basic model, data from Mar 1 to Jun 20 used for advanced model). Histories of (a) individuals in hospital (MCU and ICU), (b) individuals in ICU, (c) accumulated fatalities, and (d) fraction of infected individuals in the total population.
